# Psychoactive substances in natural and unnatural deaths in Norway and Sweden – a study on victims of suicide and accidents compared with natural deaths in psychiatric patients

**DOI:** 10.1186/s12888-019-2015-9

**Published:** 2019-01-18

**Authors:** Ida Kathrine Gravensteen, Øivind Ekeberg, Ingemar Thiblin, Karin Helweg-Larsen, Erlend Hem, Sidsel Rogde, Ingvild Maria Tøllefsen

**Affiliations:** 1 Department of Forensic Sciences, Oslo University Hospital, Box 4950 Nydalen, N-0424 Oslo, Norway; 2 Department of Behavioural Sciences in Medicine, Institute of Basic Medical Sciences, Faculty of Medicine, University of Oslo, Box 1111 Blindern, N-0317 Oslo, Norway; 3 Division of Mental Health and Addiction, Oslo University Hospital Ullevaal, Box 4956 Nydalen, N-0424 Oslo, Norway; 4 Department of Surgical Sciences, Uppsala University, Box 256, 751 05 Uppsala, Sweden; 5 Department of Social Medicine, University of Copenhagen, Copenhagen, Denmark; 6 Institute of Clinical Medicine, University of Oslo, Box 1072 Blindern, N- 0316 Oslo, Norway; 7 Division of Medicine, Department of Acute Medicine, Oslo University Hospital Ullevaal, Box 4950 Nydalen, N-0424 Oslo, Norway

**Keywords:** Accidental deaths, Natural deaths, Psychoactive substances, Suicide

## Abstract

**Background:**

The extent of post-mortem detection of specific psychoactive drugs may differ between countries, and may greatly influence the national death register’s classification of manner and cause of death. The main objective of the present study was to analyse the magnitude and pattern of post-mortem detection of various psychoactive substances by the manner of death (suicide, accidental, undetermined and natural death with a psychiatric diagnosis) in Norway and Sweden.

**Methods:**

The Cause of Death Registers in Norway and Sweden provided data on 600 deaths in 2008 from each country, of which 200 were registered as suicides, 200 as accidents or undetermined manner of death and 200 as natural deaths in individuals with a diagnosis of mental disorder as the underlying cause of death. We examined death certificates and forensic reports including toxicological analyses.

**Results:**

The detection of psychoactive substances was commonly reported in suicides (66 and 74% in Norway and Sweden respectively), accidents (85 and 66%), undetermined manner of deaths (80% in the Swedish dataset) and in natural deaths with a psychiatric diagnosis (50 and 53%). Ethanol was the most commonly reported substance in the three manners of death, except from opioids being more common in accidental deaths in the Norwegian dataset. In cases of suicide by poisoning, benzodiazepines and z-drugs were the most common substances in both countries. Heroin or morphine was the most commonly reported substance in cases of accidental death by poisoning in the Norwegian dataset, while other opioids dominated the Swedish dataset. Anti-depressants were found in 22% of the suicide cases in the Norwegian dataset and in 29% of suicide cases in the Swedish dataset.

**Conclusions:**

Psychoactive substances were detected in 66 and 74% of suicides and in 85 and 66% of accidental deaths in the Norwegian and Swedish datasets, respectively. Apart from a higher detection rate of heroin in deaths by accident in Norway than in Sweden, the pattern of detected psychoactive substances was similar in the two countries. Assessment of a suicidal motive may be hampered by the common use of psychoactive substances in suicide victims.

## Background

Substance abuse is a major public health problem and a risk factor for premature death in intentional, accidental, and natural circumstances, and can have a severe economic, emotional, and society impact on individuals, families and within communities [1–3]. In forensic medicine, determination of manner of death as intentional or accidental can be particularly challenging in deaths caused by poisoning or related to substance abuse, where there is no clear evidence for suicidal intention.. Hence, the frequency and accuracy of post-mortem examinations, including toxicological analyses, may ultimately impact death statistics [4]. In the USA, the percentage of drug poisoning classified as having undetermined manners of death range from 1 to 85% between states and specification of the drugs involved varies by type of death investigation system [5].

Few studies have systematically examined the post-mortem occurrence of psychoactive substances according to classified manner of death: natural death, accident, suicide or undetermined manner of death. In a large observational study from England on drug-related fatal injuries, the most frequent substance detected was alcohol (36%), followed by sedative-hypnotics, cannabis, heroin and cocaine. Antidepressants were more often detected in intentional fatal injuries (suicides by other methods than poisoning) compared with accidents (12% vs 2%) [6]. An important proportion of suicides is committed by mentally ill persons that are or have been treated with psychoactive prescription drugs [7–9]. Hence, it is likely that such drugs are commonly detected in this category of deaths. In a Swedish study on suicides by hanging and poisoning, paracetamol, citalopram, diazepam, sedative antihistamines, and zopiclone were amongst the top-ten drugs detected [10].

Opioid poisoning is relatively frequent among drug abusers [11], and the associated manner of death may be registered as a suicide, accident or natural death [12]. The pattern of substance abuse varies between countries. Norway reports a relatively high prevalence of intravenous drug abuse compared with the other Scandinavian countries, Sweden and Denmark [13, 14]. In all three countries, among drug abusers, poisoning by opioids is reported as the main cause of death, with methadone more commonly detected in deaths in Denmark and Sweden, and heroin/morphine in Norway [14]. Regional differences in substance abuse may reflect general availability, but aside from opioid overdoses, less is known about patterns of toxicological findings in the general population of suicides and accidents in Scandinavia. Since a peak in 2001, there has been no definite decline in deaths due to accidental poisoning in Norway [15]. According to the Swedish official death statistics there has been a gradual increase of drug related deaths with some fluctuations during the past five decades and a marked increase during the period 2006–2014 [16]. In the latter period, the rate of drug related deaths increased from 3.6 to 8.1 per 100.000 inhabitants per year, and most of the deaths involved opioid overdose [14].

In order to enable societies to implement relevant prevention strategies to reduce premature deaths, increased knowledge about related substance abuse patterns is necessary. The main purpose of this study was to analyse the occurrence and pattern of various psychoactive substances in a population of suicides and accidents as well as in natural deaths with a psychiatric diagnosis. In addition to describe patterns of substance findings according to manner of death, and deaths due to acute poisoning versus other methods, we aimed to compare datasets from two neighbouring countries; Norway and Sweden.

## Methods

### Description of data

This study includes data of the deaths of 1200 persons aged 18 years and older, 600 from Norway and 600 from Sweden from the year 2008. It follows a previous assessment of the reliability of suicide statistics in Scandinavia [17].

From the Cause of Death Registers for each country, we retrieved data on 200 deaths registered as suicides, 200 as accidents or undetermined manner of death and 200 as natural deaths (i.e. death as a result of a disease or an internal malfunction of the body not caused by external forces or poisoning). The suicide sample included suicide by all methods (ICD-10: X60–X84, Y870). The accidents and undetermined manners of death covered traffic accidents (ICD-10: V43–V45.5, V47–V48.5, V49.4), accidental poisoning (ICD-10: X40–X49), accidental drowning (ICD-10: W65–W74), accidental fire and flame (ICD-10: X00–X09) and undetermined intent (ICD-10: Y10–Y34, Y872). The Norwegian dataset included no cases of undetermined intent, as no deaths were registered under this category in the Norwegian Cause of Death Register in 2008.

Since we hypothesised that psychoactive substances would also be commonly detected among forensically examined natural deaths with mental illness as the underlying cause of death, this group were also included for comparative purposes. These included ‘mental and behavioural disorders due to psychoactive substance use’ (ICD-10: F10–F19), ‘schizophrenia, schizotypal and delusional disorders’ (ICD-10: F20–F29), ‘mood (affective) disorders’ (ICD-10: F30–F39) and ‘disorders of adult personality and behaviour’ (ICD-10: F60–F69).

### Material

In Norway and Sweden, it is mandatory for physicians to report all suspected unnatural deaths to the police, who then decide whether a forensic autopsy shall be performed. In Norway, only some of the centres that perform forensic autopsies submit a complete autopsy report to the Norwegian Cause of Death Register, whereas the others submit a revised death certificate. In the Norwegian sample of 600 deaths, forensic autopsies had been performed in 325 (54%) of cases, and for 266 of these, a complete autopsy report was available. For the remaining 59 cases, revised death certificates were available. In the Swedish dataset, a forensic autopsy had been performed in 483 (81%) of the 600 cases, a complete autopsy report was available for 469 cases and we used revised death certificates for the remaining 14 (Table 1). A more detailed description of the data material and study population has been published previously [17].

**Table 1:** Sample characteristics

	Total	Norway	Sweden
Total number of cases (n)	1200	600	600
Natural deaths		200	200
Suicides		200	200
Accidents		200	140
Undetermined manner of death		0	60
Performed autopsy (n)	808 (67%)	325 (54%)	483 (81%)
Natural deaths		32 (16%)	108 (54%)
Suicides		136 (68%)	192 (96%)
Accidents		157 (79%)	124 (89%)
Undetermined manner of death			59 (98%)

For the forensic autopsy cases, accounts of psychoactive substance findings were based on information given on the revised death certificates or in the autopsy report. However, for the Norwegian cases in particular, the content of the autopsy reports and revised death certificates differed in their levels of detail regarding results of the toxicological analyses. In deaths due to causes other than poisoning, results of possible toxicological analyses were not always stated. Further, in some deaths due to poisoning (suicide or accident), only the main agent was stated, while other toxicological results were probably omitted. A complete statement on toxicological findings outlining both positive and negative results was available for 233 of the Norwegian cases (39%) and 449 (75%) of the Swedish cases.

### Toxicological analyses

Detected psychoactive substances in blood, urine, other body fluids, muscle or hair were classified as alcohols (ethanol or toxic alcohols), opioids (heroin/morphine or other opioids), cannabinoids, psychostimulants (amphetamine/methamphetamine, cocaine/benzoylecgonine, ecstasy, 3-methoxy-4-methylamphetamine and related substances), benzodiazepines and z-drugs (zolpidem and zopiclone), sedative antihistamines (alimemazine, promethazine, dexchlorpheniramine, hydroxyzine and related substances), anti-depressants (selective serotonin reuptake inhibitors [SSRIs], serotonin and noradrenaline reuptake inhibitors, and other anti-depressants), antipsychotics, antiepileptics and other or unspecified psychoactive substances. Metabolites were classified accordingly. In morphine positive cases, low concentrations of codeine were assumed to be derived from intake of heroin/morphine. The detection of ethanol confined to urine was most likely a post-mortem occurrence and was not included in the analyses.

### Statistics

We used the Statistical Package for Social Science (version 23.0; SPSS Inc., Armonk, NY, USA). We present estimates of post-mortem forensic findings by the manner of death (suicide, accidental, undetermined and natural death) in the Norwegian and Swedish datasets as numbers and percentages. Proportions of detected substances were compared between suicides and accidents in each country using chi-squared tests. Chi-squared tests were also used to compare the frequency of psychoactive substances in suicides/accidents by poisoning between the Norwegian and Swedish datasets. Two-sided *P*-values < 0.05 were regarded as statistically significant.

## Results

### Psychoactive substances in suicides, accidents and natural deaths

Post-mortem detection of psychoactive substances was commonly reported for suicides (66 and 74%), accidents (85 and 66%), undetermined manner of death (80%, Swedish dataset only) as well as in natural deaths with a psychiatric diagnosis (50 and 53%) in the Norwegian and Swedish datasets, respectively (Table 2). In both countries and in all manners of death, detection of ethanol was most frequent (27–38%), with the exception of accidents in the Norwegian dataset, where opioids were the most frequent (55%). In both the Norwegian and Swedish datasets, opioids, cannabinoids and psychostimulants were more frequently reported in accidents than in suicides (*P* < 0.05). Anti-depressants were detected in 22 and 29% of the suicides in the Norwegian and Swedish datasets, respectively. Among undetermined deaths in the Swedish dataset, anti-depressants (37%) and benzodiazepines (37%) were the most frequently reported substances, and opioids (34%) and ethanol (34%) the second. In natural deaths with a psychiatric diagnosis, the detection of psychoactive substances other than ethanol was relatively rare in both countries.

**Table 2:** Detection of psychoactive substances in all deaths, n (%)

	Norway				Sweden				
	Natural deaths *n* = 32	Suicides *n* = 136	Accidents *n* = 157	P-value (×2)	Natural deaths *n* = 108	Suicides *n* = 192	Accidents *n* = 124	Undetermined deaths *n* = 59	*P*-value (× 2)
Alcohols	10 (31)	36 (27)	49 (31)^o^	0.650	37 (34)	73 (38)	44 (36) o	20 (34)	0.894
Ethanol	10 (31)	36 (27)	47 (30)		37 (34)	73 (38)	44 (36)	20 (34)	
Toxic alcohols^a^	0	1 (1)	2 (1)		0	0	0	0	
Opioids	1 (3)	16 (12)	87 (55)^***^	< 0.001	10 (9)	27 (14)	38 (31)***	20 (34)	< 0.001
Heroin/morphine	0	7 (5)	65 (41)		2 (2)	4 (2)	13 (11)	6 (10)	
Other opioids^b^	1 (3)	11 (8)	30 (19)		9 (8)	25 (13)	29 (23)	18 (31)	
Cannabinoids	2 (6)	6 (4)	20 (13)^*^	0.036	4 (4)	3 (2)	10 (8)**	3 (5)	0.042
Psychostimulants^c^	1 (3)	10 (7)	24 (15)^*^	0.031	3 (3)	6 (3)	15 (12)**	4 (7)	0.004
Benzodiazepines and z-drugs^d^	3 (9)	46 (34)	64 (41)^o^	0.003	12 (11)	49 (26)	30 (24)^o^	22 (37)	0.001
Sedative antihistamines^e^	1 (3)	9 (7)	8 (5)^o^	0.698	8 (7)	33 (17)	14 (11)^o^	14 (24)	0.013
Antidepressants	3 (9)	30 (22)	23 (15)^o^	0.114	7 (7)	56 (29)	21 (17)^*^	22 (37)	< 0.001
SSRI/SNRI	3 (9)	21 (15)	15 (10)		6 (6)	50 (26)	17 (14)	19 (32)	
Other antidepressants	0	12 (9)	12 (8)		1 (1)	16 (8)	8 (7)	5 (9)	
Antipsychotics	2 (6)	16 (12)	12 (8)^o^	0.396	1 (1)	6 (3)	5 (4)^o^	2 (3)	0.543
Antiepileptics^f^	0	7 (5)	10 (6)^o^	0.336	7 (7)	11 (6)	4 (3)^o^	2 (3)	0.600
Other/unspecified psychoactive agents^g^	3 (9)	12 (9)	19 (12)^o^	0.644	1 (1)	5 (3)	1 (1)^o^	3 (5)	0.205
Any psychoactive agent	16 (50)	90 (66)	134 (85)***	< 0.001	57 (53)	142 (74)	82 (66)^o^	47 (80)	< 0.001

^a^Methanol, isopropanolol

^b^Methadone, buphrenorphine, oxycodone, ketobimedone, tramadol, fentanyl, dextropropoxyphene. Includes combination drugs containing codeine, but not codeine assumed to be derived from heroin/morphine

^c^Amphetamine/methamphetamine, cocaine/benzoylecgonine, MMA, ecstasy and related substances

^d^Zolpidem and zopiclone

^e^Alimemazine, promethazine, dexchlorfeniramine, hydroxyzine and related substances

^f^Karbamazepin, lamotrigin, fenobarbital, gabapentin, pregabalin, fenytoin, topiramat (not benzodiazepines)

^g^GHB, nimbex, tiopental, baclofen, propofol, ketamin, butan, orfenadrin, somadril/karsiprodol, biperiden, kloroxazon

^o^Accidents not significantly different from suicides (*p*-value > 0.05)

*Accidents significantly different from suicides (*p*-value < 0.05)

**Accidents significantly different from suicides (*p*-value < 0.01)

***Accidents significantly different from suicides (*p*-value < 0.001)

### Suicides and accidents by poisoning

Among suicides by poisoning, benzodiazepines and z-drugs were the most commonly detected psychoactive substances in the Norwegian (62%) and Swedish (46%) datasets (Tables 3 and 4). Ethanol, anti-depressants and opioids were also common in this category of deaths. Detection of antipsychotic drugs and sedative antihistamines differed in the two datasets. Antipsychotic drugs were detected in 24% of suicides by poisoning in the Norwegian dataset but in only 7% of the Swedish dataset (*P* < 0.05). Among suicides by poisoning in the Swedish dataset, sedative antihistamines were the second most common substance group (41%) but were detected in only 11% of the Norwegian cases (*P* < 0.01). Detection of psychostimulants such as amphetamine/methamphetamine and cocaine were more common among poisoning suicides in Norway (14%) compared with Sweden (2%).

**Table 3:** Psychoactive substances in deaths by poisoning and all other causes, according to manner of death, n (%)

	Norway				Sweden					
	Suicides		Accidents		Suicides		Accidents		Undetermined deaths	
	Poisoning *n* = 37	Other *n* = 99	Poisoning *n* = 112	Other *n* = 45	Poisoning *n* = 56	Other *n* = 136	Poisoning *n* = 62	Other *n* = 62	Poisoning *n* = 33	Other *n* = 26
Ethanol	14 (38)	22 (22)	32 (29)^o^	15 (33)	22 (39)	51 (38)	27 (44)^o^	17 (27)	11 (33)^o^	9 (35)
Opioids	12 (32)	4 (4)	82 (73)^***^	5 (11)	19 (34)	8 (6)	35 (57)^*^	3 (5)	19 (58)*	1 (4)
Heroin/morphine	4 (11)	3 (3)	63 (56)	2 (4)	3 (5)	1 (1)	13 (21)	0	6 (18)	0
Other opioids^a^	9 (24)	2 (2)	26 (23)	4 (9)	18 (32)	7 (5)	26 (42)	3 (5)	17 (52)	1 (4)
Psychostimulants^b^	5 (14)	5 (5)	23 (21)^o^	1 (2)	1 (2)	5 (4)	14 (23)^**^	1 (2)	3 (9)^o^	1 (4)
Cannabinoids	0	6 (6)	16 (14)^*^	4 (9)	2 (4)	1 (1)	10 (16)^*^	0	3 (9)^o^	0
Benzodiazepines and z-drugs^c^	23 (62)	23 (23)	57 (51)^o^	7 (16)	26 (46)	23 (17)	27 (44)^o^	3 (5)	19 (58)^o^	3 (12)
Sedative antihistamines^d^	4 (11)	5 (5)	8 (7)^o^	0	23 (41)	10 (7)	12 (19)^*^	2 (3)	13 (39)^o^	1 (4)
Antidepressants	13 (35)	17 (17)	20 (18)^*^	3 (7)	23 (41)	33 (24)	17 (27)^o^	4 (7)	18 (55)^o^	4 (15)
SSRI/SNRI	8 (22)	13 (13)	13 (12)	2 (4	18 (32)	32 (24)	14 (23)	3 (5)	15 (46)	4 (15)
Other antidepressants	7 (19)	5 (5)	10 (9)	2 (4)	12 (21)	4 (3)	6 (10)	2 (3)	5 (15)	0
Antipsychotics	9 (24)	7 (7)	11 (10)^*^	1 (2)	4 (7)	2 (2)	5 (8)^o^	0	2 (6)^o^	0
Antiepileptics^e^	6 (16)	1 (1)	9 (8) ^o^	1 (2)	7 (13)	4 (3)	3 (5)^o^	1 (2)	2 (6)^o^	0
Other/unspecified psychoactive agent^f^	4 (11)	8 (8)	18 (16)^o^	1 (2)	3 (5)	2 (2)	1 (2)^o^	0	3 (9)^o^	0
Any psychoactive agent	32 (87)	58 (59)	108 (96)*	26 (58)	55 (98)	87 (64)	59 (95)^o^	23 (37)	33 (100)^o^	14 (54)

^a^Methadone, buphrenorphine, oxycodone, ketobimedone, tramadol, fentanyl, dextropropoxyphene. Includes combination drugs containing codeine, but not codeine assumed to be derived from heroin/morphine

^b^Amphetamine/methamphetamine, cocaine/benzoylecgonine, MMA, ecstasy and related substances

^c^Zolpidem and zopiclone

^d^Alimemazine, promethazine, dexchlorfeniramine, hydroxyzine and related substances

^e^Karbamazepin, lamotrigin, fenobarbital, gabapentin, pregabalin, fenytoin, topiramat (not benzodiazepinesf GHB, nimbex, tiopental, baclofen, propofol, ketamin, butan, orfenadrin, somadril/karsiprodol, biperiden, kloroxazon

^f^GHB, nimbex, tiopental, baclofen, propofol, ketamin, butan, orfenadrin, somadril/karsiprodol, biperiden, kloroxazon

^o^Accidents/undetermined deaths by poisoning not significantly different from suicides by poisoning (*p*-value > 0.05)

*Accidents/undetermined deaths by poisoning not significantly different from suicides by poisoning (p-value < 0.05)

**Accidents/undetermined deaths by poisoning not significantly different from suicides by poisoning (p-value < 0.01)

***Accidents/undetermined deaths by poisoning not significantly different from suicides by poisoning (p-value < 0.001)

**Table 4:** Psychoactive substances according to suicide method, n (%)

	Intentional self-poisoning ICD-10: X60-X69		Intentional self-harm by hanging, strangulation and suffocation. ICD-10: X70		Other intentional self-harm ICD-10: X71-X75, X78, X80-X84, X870	
	Norway n = 37	Sweden n = 56	Norway *n* = 44	Sweden *n* = 67	Norway *n* = 55	Sweden *n* = 69
Ethanol	14 (38)	22 (39) o	10 (23)	26 (39)	12 (22)	25 (36)
Opioids	12 (32)	19 (34) o	3 (7)	2 (3)	1 (2)	6 (9)
Heroin/morphine	4 (11)	3 (5)	2 (5)	0	1 (2)	1 (1)
Other opioidsa	9 (24)	18 (32)	2 (5)	2 (3)	0	5 (7)
Cannabinoids	0	2 (4)^o^	4 (9)	1 (2)	2 (4)	0
Psychostimulantsb	5 (14)	1 (2)^*^	5 (11)	3 (5)	0	2 (3)
Benzodiazepines, z-drugsc	23 (62)	26 (46)^o^	12 (27)	13 (19)	11 (20)	10 (15)
Sedative antihistaminesd	4 (11)	23 (41)^**^	3 (7)	4 (6)	2 (4)	6 (9)
Antidepressants	13 (35)	23 (41)^o^	8 (18)	16 (24)	9 (16)	17 (25)
SSRI/SNRI	8 (22)	18 (32)	6 (14)	16 (24)	7 (13)	16 (23)
Other antidepressants	7 (19)	12 (21)	2 (5)	1 (2)	3 (6)	4 (6)
Antipsychotics	9 (24)	4 (7)^*^	3 (7)	1 (2)	4 (7)	1 (1)
Antiepilepticse	6 (16)	7 (13)^o^	1 (2)	3 (5)	0	1 (1)
Other/unspecified psychoactive agentf	4 (11)	3 (5)^o^	2 (5)	1 (2)	6 (11)	1 (1)
Any psychoactive agent	32 (87)	55 (98)*	26 (59)	41 (61)	32 (58)	46 (67)

^a^Methadone, buphrenorphine, oxycodone, ketobimedone, tramadol, fentanyl, dextropropoxyphene. Includes combination drugs containing codeine, but not codeine assumed to be derived from heroin/morphine

^b^Amphetamine/methamphetamine, cocaine/benzoylecgonine, MMA, ecstasy and related substances

^c^Zolpidem and zopiclone

^d^Alimemazine, promethazine, dexchlorfeniramine, hydroxyzine and related substances

^e^Karbamazepin, lamotrigin, fenobarbital, gabapentin, pregabalin, fenytoin, topiramat (not benzodiazepinesf GHB, nimbex, tiopental, baclofen, propofol, ketamin, butan, orfenadrin, somadril/karsiprodol, biperiden, kloroxazon

^f^GHB, nimbex, tiopental, baclofen, propofol, ketamin, butan, orfenadrin, somadril/karsiprodol, biperiden, kloroxazon

^o^No significant differences in suicides by poisoning in Norway and Sweden (p-value > 0.05)

*Significant differences in suicides by poisoning in Norway and Sweden (p-value < 0.05)

**Significant differences in suicides by poisoning in Norway and Sweden (p-value < 0.01)

***Significant differences in suicides by poisoning in Norway and Sweden (p-value < 0.00)

In both datasets, among accidents by poisoning, opioids were the most commonly detected psychoactive substance (Table 3), with a significantly higher prevalence in the Norwegian dataset (73%) than in the Swedish dataset (57%; *P* < 0.05). Of the opioids, heroin/morphine was more frequent in the Norwegian dataset (56% vs. 21%; *P* < 0.001), while other opioids were more frequent in the Swedish dataset (23% vs. 42%; *P* < 0.05). In both countries, benzodiazepines, z-drugs, ethanol, psychostimulants and anti-depressants were also commonly detected in accidents by poisoning. Ethanol (29% vs. 44%; *P* < 0.05) and sedative antihistamines (7% vs. 19%; *P* < 0.05) were more frequently detected in the Swedish dataset of accidents by poisoning.

### Suicides and accidents by methods other than poisoning

The detection of psychoactive substances was also common in suicides and accidents not caused by poisoning (Tables 3 and 4). Among suicides by methods other than poisoning, psychoactive substances were detected in 59% of the Norwegian dataset and 64% of the Swedish dataset. Among accidents other than by poisoning, psychoactive substances were reported in 58% of the Norwegian dataset and in 37% of the Swedish dataset. Ethanol was reported in 22% of Norwegian and 38% of Swedish datasets for ‘non-poisoning’ suicides, and in 33 and 27% of ‘non-poisoning’ accidents, respectively.

## Discussion

This study confirms a high frequency of psychoactive substance detection in post-mortem examinations of deaths classified as suicides or accidents as reported by previous studies [18–23]. Substances other than ethanol were less commonly detected in natural deaths with a psychiatric diagnosis.

In accidental deaths by poisoning, opioids were the most commonly reported substances in both the Norwegian and Swedish datasets. However, while heroin and morphine dominated in the Norwegian dataset, other opioids dominated in the Swedish dataset. These findings are in accordance with those of previous studies [14, 24] and probably reflect national differences in the availability of illicit and prescription opioids in the two countries. In the recent years, the general tendency in the Scandinavian countries has been that medicinal opioids are replacing heroin, possibly connected to a general decline in heroin availability around 2010/2011 [25].

Furthermore, strong opioids (morphine, fentanyl and oxycodone) are also becoming more frequently prescribed for pain in Sweden [26] and the use of opioids in opioid agonist therapy for problematic drug users is increasing as an effect of the less rigorous regulations introduced in 2005 [27]. According to a recent Swedish study, 24% of 400 patients in opioid agonist therapy reported that they had diverted methadone or buprenorphine to other people “in the last month” [28]. Similarly, a Finnish study found that 20% of patients gave away or sold their doses [29].

Even though the suicide statistics in Scandinavia has been found to be quite reliable [17], the state of mind of the suicide victims may be difficult to assess when so many were under the influence of psychoactive substances at the time of death.

In suicide victims, the prevalence of ethanol was similar to that found in previous studies in Norway and Sweden [11–13], and was relatively high (22–39%) regardless of suicide method. In a Polish survey, suicide under the influence of alcohol was strongly associated with alcohol dependence [30], emphasizing the importance of suicidal risk assessment in healthcare for this patient group.

More than half of all people who die by suicide meet criteria for current depressive disorder [31]. There is strong evidence that anti-depressant use and psychotherapy may prevent or reduce affective mental disorders [32]. Anti-depressants were detected in only 22% of Norwegian and 29% of Swedish cases classified as suicides in the present datasets, and antipsychotics were detected in only 12 and 3% of the cases, respectively. This might indicate either low detection or diagnosis of mental diseases, an insufficient prescription of relevant drugs, or low compliance with regard to the medical treatment of psychiatric disorders in the population at risk. In other terms, low detection of anti-depressants among suicide victims may reflect a potential to reduce suicide rates through improvements in treatment. However, there is conflicting evidence about a potential correlation between the use of anti-depressants and suicide rates. Sales figures of SSRIs in Norway and Sweden had a fivefold increase in 1990–2006, but no inverse relationship between the increase in sales of SSRIs and declining suicide rates has been found [33]. A Finnish study of suicide among those with major depression reported that only 3% had received anti-depressants in an adequate (therapeutic) dosage, and none of those with severe psychoses had received adequate psychopharmacological treatment [34].

In the Swedish dataset, sedative antihistamines were the second most commonly detected substance in suicides by poisoning and were also commonly detected in accidental poisoning cases, whereas in the Norwegian dataset the prevalence of benzodiazepines and z-drugs was higher. This indicates that for sleep disorders, antihistamines are more commonly prescribed than benzodiazepines and z-drugs in Sweden compared with Norway.

Anti-depressants, benzodiazepines, opioids and ethanol were detected in about one-third of the Swedish cases classified as undetermined manner of death. The pattern of psychoactive substances in this classification differed from that found in suicides, accidental deaths and natural deaths, implying the complexity of this group.

While the prevalence of opioids among undetermined deaths was similar to accidents, detection of prescription drugs such as sedative antihistamines and anti-depressants was higher and comparable to suicides. In a previous study, conducted by our group, 21% of the undetermined deaths in the Swedish data set were reclassified as suicides, while the great majority was reclassified as accidents [17].

### Strengths and limitations

Recent toxicological studies in suicides are limited to single cities or a small geographical or societal region [35–37]. To our knowledge, this is the first study that has included nationwide data from two Scandinavian countries, which allowed transnational comparison of patterns of toxicological findings in suicides, accidental deaths, natural deaths with a psychiatric diagnosis and cases with an undetermined intent.

The present study is based on datasets comprising a large proportion of cases within the extracted categories, including adults of both genders in a nationwide sample comparable to the official mortality statistics. The autopsy rates in the two datasets corresponded with national death statistics [38, 39] and we consider the internal validity of the study to be reasonable.

The study had some limitations. We might have missed results of toxicological analyses that were not included in the available autopsy reports or revised death certificates. Furthermore, the relatively low autopsy rate in Norway may limit the reliability of the rate of detected psychoactive drugs and be responsible for the under-reporting of substances not directly related to the cause of death. It may even impact the validity of manner of death classification, possibly resulting in a lower official suicide rate [40].

### Implications and further research

Standards for post-mortem examinations and in particular for toxicological analyses can give important information in order to establish the cause of death. Mortality data is an essential tool in transnational analyses that might identify preventable risk factors for premature deaths and guide public health efforts to prevent those deaths. The differences between Norway and Sweden in the frequency of forensic autopsies and reporting of toxicological findings, may affect cause as well as manner of death classification. In Norway, only a small proportion (16%) of deaths classified as natural deaths with a psychiatric diagnosis underwent autopsy, and there may be a risk of misclassified deaths by poisoning in this group.

A higher rate of forensic autopsies, including standardized toxicological analyses, would result in more reliable data on the presence of toxic agents, in cases of sudden death with sparse clinical information in particular, and in Norway in general. Better information on the related circumstances, including the clinical history of the deceased, is needed for a more reliable assessment of the manner of death. In addition, information about the history of substance abuse and ingestion of toxic agents before death may give a broader basis for the detection of psychoactive substances.

The relatively low detection rate of anti-depressants in deaths by suicide could indicate low compliance and/or low treatment frequency of affective mental disorders in Sweden and Norway, but this should be further assessed in future research.

## Conclusions

In a population sample based on forensic autopsies from Norway and Sweden, post-mortem detection of psychoactive substances was reported in more than two-thirds of suicides and accidents, and in about half of natural deaths in patients with a psychiatric diagnosis.

National differences in the pattern of detected psychoactive substances existed. Ethanol was detected in about one third of all deaths in each manner category, and was the most commonly detected drug, except in accidental deaths in the Norwegian dataset, where opioids were most common. In both countries, detection of benzodiazepines and z-drugs was common in suicides by poisoning. In the Swedish dataset, sedative antihistamines were almost as frequently detected as other tranquillizers. Among accidental deaths by poisoning in the Norwegian dataset, heroin or morphine was the most commonly detected drug, while other opioids dominated in the Swedish dataset.

Anti-depressants were detected in 22% of the suicides in the Norwegian dataset and in 29% in the Swedish dataset. Other studies estimate that about 40–60% of suicide victims suffered from depression, and thus would be candidates for the prescription of anti-depressants; thus, the reason for the relatively low detection of anti-depressants among the suicide cases in our study is unclear.

## Abbreviations

SSRI: Selective serotonin reuptake inhibitors
